# Characterization and Quantification of Innate Lymphoid Cell Subsets in Human Lung

**DOI:** 10.1371/journal.pone.0145961

**Published:** 2016-01-04

**Authors:** Katrien C. De Grove, Sharen Provoost, Fien M. Verhamme, Ken R. Bracke, Guy F. Joos, Tania Maes, Guy G. Brusselle

**Affiliations:** Department of Respiratory Medicine, Laboratory for Translational Research in Obstructive Pulmonary Diseases, Ghent University Hospital, Ghent, Belgium; Harvard Medical School, UNITED STATES

## Abstract

**Background:**

Innate lymphoid cells (ILC) are a new family of innate immune cells that have emerged as important regulators of tissue homeostasis and inflammation. However, limited data are available concerning the relative abundance and characteristics of ILC in the human lung.

**Methods:**

The aim of this study was to characterize and enumerate the different ILC subsets in human lung by multi-color flow cytometry.

**Results:**

Within the CD45^+^ Lin^-^ CD127^+^ pulmonary ILC population, we identified group 1 (ILC1), group 2 (ILC2) and group 3 (ILC3) innate lymphoid cells using specific surface markers (i.e. IL12Rβ2, CRTH2 and CD117 respectively) and key transcription factors (i.e. T-bet, GATA-3 and RORγT respectively). Based on the presence of NKp44, ILC3 were further subdivided in natural cytotoxicity receptor (NCR)^+^ and NCR^-^ ILC3. In addition, we demonstrated the production of signature cytokines IFN-γ, IL-5, IL-17A, IL-22 and GM-CSF in the pulmonary ILC population. Interestingly, we observed a tendency to a higher frequency of NCR^-^ ILC3 in lungs of patients with chronic obstructive pulmonary disease (COPD) compared with controls.

**Conclusions:**

We show that the three main ILC subsets are present in human lung. Importantly, the relative abundance of ILC subsets tended to change in COPD patients in comparison to control individuals.

## Introduction

Innate lymphoid cells (ILC) are a newly characterized heterogeneous family of the innate immune system, which have emerged as important regulators of tissue homeostasis, immunity and inflammation. ILC are morphologically similar to their counterpart of the adaptive immune system, T- and B-cells. Although they lack specific rearranged antigen receptors, which is a hallmark of the adaptive immune system, these innate immune cells can produce an array of cytokines in response to various danger signals and changes in homeostasis [1,2]. Analogous with T-cells, ILC have been classified into three subsets – group 1 (ILC1), group 2 (ILC2) and group 3 (ILC3) innate lymphoid cells – depending on their phenotype, function and transcriptional regulation. The ILC1 subset includes both natural killer (NK) cells and non-toxic ILC1. These non-toxic ILC1 are characterized by the expression of the transcription factor T-bet and production of IFN-γ in response to interleukin (IL)-12. ILC2 depend on the transcription factor GATA-3 for their function and development and produce the type 2 cytokines, IL-5 and IL-13, in response to IL-25 and IL-33. Finally, the ILC3 subset is divided into natural cytotoxicity receptor (NCR, i.e. NKp44, NKp46 and NKp30)^+^ ILC3 and NCR^-^ ILC3. The latter group is a heterogeneous population which also encompasses lymphoid tissue inducers (LTi) cells. These ILC3 express the transcription factor RORγT and are capable to produce IL-17A, IL-22 or granulocyte-macrophage colony-stimulating factor (GM-CSF) in response to IL-23 and IL-1β [3,4].

Over the last few years, human ILC subsets have been studied in several tissues, including the skin and intestines [4]. To characterize these ILC, several surface markers have been proposed based on studies in the gut [3]. To our knowledge, research into ILC in the human respiratory system is currently limited to ILC2 [5–10]. Recently, one study showed the presence of ILC subsets in human lung tissue in the context of lung cancer [11]. However, the relative abundance of ILC1, NCR^+^ ILC3 and NCR^-^ ILC3 in human lung tissue under inflammatory conditions such as chronic obstructive pulmonary disease (COPD) has not yet been characterized. COPD is a chronic inflammatory lung disease that is associated with the development of emphysema and lymphoid follicles. It has been shown that besides the adaptive immune system also innate immune cells substantially contribute to the pathogenesis of COPD [12].

In this manuscript, we identified and quantified non-toxic ILC1, ILC2, NCR^+^ ILC3 and NCR^-^ ILC3 subsets in human lung by flow cytometry based on several phenotypical markers and signature transcription factors. Further, we examined the expression of specific cytokines in the pulmonary ILC population. Finally, we compared the relative abundance of the ILC subsets in control subjects and patients with COPD.

## Materials and Methods

### Lung tissue

Lung tissue was obtained from patients who underwent a surgical lung resection at Ghent University Hospital for solitary pulmonary tumors. Tissue was collected by a pathologist at maximum distance from the lung lesion, and showed no signs of retro-obstructive pneumonia or tumor invasion. Sixteen subjects were enrolled in our study and classified into 2 groups: 5 control subjects and 11 patients with COPD stage I or II. COPD diagnosis and severity was defined using pre-operative spirometry according to the Global Initiative for Chronic Obstructive Lung Disease (GOLD) classification: all control subjects had a post-bronchodilator ratio of forced expiratory volume in 1 second (FEV_1_) to forced vital capacity (FVC) above 70%, whereas all COPD patients had a FEV_1_/FVC ratio below 70%. Patients were diagnosed with COPD GOLD I when FEV_1_≥ 80%, patients with COPD GOLD II had a FEV_1_ between 50 and 80% predicted [13]. Written informed consents were obtained from all subjects, according to the protocol approved by the medical ethical committee of Ghent University Hospital. Patient characteristics are shown in Table 1.

**Table 1:** Subject Characteristics.

Characteristics	Controls (n = 5)	COPD (n = 11)
**Gender (male/female)**	3/2	10/1
**Age, years^1^**	60 ± 11	67 ± 9
**BMI^1^^,^^2^**	25 ± 5	26 ± 5
**Smoking history, Pack Years^1^**	8 ± 13	36 ± 11
**Smoking status (never/current/ex smoker)**	3/2/0	0/5/6
**COPD GOLD Stage**	na^3^	I/II
**FEV_1_ % predicted, post-bronchodilator^1^^,^^4^**	99 ± 9	89 ± 16
**FVC % predicted, post-bronchodilator^1^^,^^5^**	107 ± 12	102 ± 18
**FEV_1_/FVC, post-bronchodilator^1^^,^^4^^,^^5^**	77 ± 5	64 ± 5

1 Data are expressed as mean ± standard deviation.

2 BMI: body mass index.

3 na: not applicable.

4 FEV_1_: forced expiratory volume in 1 s.

5 FVC: forced vital capacity.

### Single cell suspensions from lung tissue

Lung resection specimens were processed as described previously to obtain single cell suspensions [14]. In brief, lung tissue was rinsed with physiological water (0.9% NaCl) to remove residual blood. The lung tissue was cut into fine pieces and digested for 45 minutes at 37°C in digestion medium (Roswell Park Memorial Institute medium (RPMI) 1640 supplemented with 5% fetal calf serum (FCS), 2 mM L-glutamine, 0.05 mM 2-mercaptomethanol (all Invitrogen), 100 U/ml penicillin, 100 mg/ml streptomycin (Sigma-Aldrich), 1 mg/ml collagenase type 2 (Worthington Biochemical), and 0.02 mg/ml DNase I (grade II from bovine pancreas; Boehringer Ingelheim)). Cells were resuspended in 10 mM ethylenediaminetetraacetic acid (EDTA) for 5 minutes at room temperature on a shaker. Next, cells were filtered through a 40-μm cell strainer and mononuclear cells were isolated with Ficoll-Paque^TM^ plus (GE Healthcare). Finally, cells were subjected to red blood cell lysis (S1 Fig).

### Flow cytometry

Single cell suspensions were pre-incubated with human IgG to reduce nonspecific binding. For surface staining, the following human monoclonal antibodies were used: fluorescein isothiocyanate (FITC)-conjugated anti-CD45 (HI30), peridinin chlorophyll protein-cyanine 5.5 (PerCP)-conjugated anti-CD3 (OKT3), anti-CD19 (HIB19), anti-CD11c (3.9), anti-CD11b (M1/70), allophycocyanin (APC)-conjugated anti-NKp44 (P44-8), PE/indotricarbocyanine (Cy7)-conjugated anti-CD117 (104D2), brilliant violet 421^TM^-conjugated anti-CD127 (A019D5), brilliant violet 605^TM^-conjugated anti-CD56 (HCD56) (all from Biolegend); phycoerythrin (PE)-conjugated anti-IL12Rβ2 (305719; R&D systems), biotinylated anti-CRTH2 (BM19; Miltenyi Biotec) in combination with streptavidin (SAV)-APC or SAV-APC-Cy7 (BD Biosciences). For cytoplasmatic cytokine staining, cells were simulated for 15 hours with phorbol myristate acetate (PMA) and ionomycin, supplemented with brefeldin A and monensin (eBioscience) at 37°C. The intracellular fixation and permeabilization buffer set (eBioscience) was used for fixation and cell permeabilization. The following antibodies were used: PE-conjugated anti-IL-17A (eBio64CAP17), anti-IFN-γ (4S.B3), anti-IL-22 (22URTI), anti-IL-5 (TRFK5), anti-GM-CSF (GM2F3), and isotype-matched control antibodies (all eBioscience). For nuclear staining, Foxp3/transcription factor staining buffer (eBioscience) set was used in combination with the following antibodies: PE-conjugated anti-T-bet (eBio4B10), anti-GATA-3 (TWAJ), anti-RORγT (AFKJS-9), and isotype-matched control antibodies (all eBioscience).

### Data acquisition and analysis

Data acquisition was performed on a LSRFortessa running DiVa software (BD Biosciences). Cell subsets were analyzed using FlowJo Software. Statistical analysis was performed with SPSS, version 22.0 (SPSS, Chicago, USA). A non-parametric test (Mann-Whitney-U) was used to compare the different ILC subsets in the control versus COPD group, according to the standard statistical criteria. P-values < 0.05 were considered as significant. Patient characteristics are expressed as the mean ± standard deviation (SD). Flow cytometric data are expressed as the mean ± standard error of the mean (SEM).

## Results

### Characterization of innate lymphoid cell subsets in human lung

**Fig 1:**
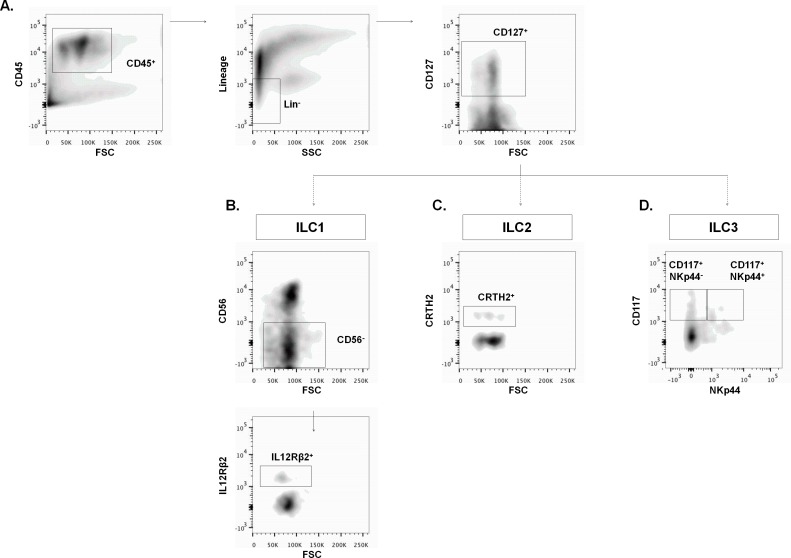
Characterization of innate lymphoid cell subsets in human lung. ILC subsets were identified by flow cytometry on single cell suspensions of digested human lung (n = 16). Fluorescence-minus-one controls were used to set gates. **A**, The pulmonary ILC population was characterized as CD45^+^, Lin^-^ (i.e. CD3, CD19, CD11c, CD11b) and CD127^+^ cells. Subsets were further defined based on specific surface markers. **B**, Non-toxic ILC1 were further gated as CD56^-^, IL12Rβ2^+^ cells. **C**, The ILC2 subset expresses CRTH2. **D,** NCR^+^ ILC3 were CD117^+^, NKp44^+^, whereas NCR^-^ ILC3 were characterized as CD117^+^, NKp44^-^ cells.

In humans, the presence of ILC subsets has been studied in blood, gut and skin. In order to characterize ILC subsets in human lung tissue, we analyzed surgical lung resection specimens (n = 16; patient characteristics are shown in Table 1). Single cell suspensions were stained to identify the different ILC subsets. Fluorescence-minus-one controls were used to set flow cytometric gates (S2 Fig). ILC were characterized as CD45^+^ cells, a marker that is present on all hematopoietic cells. In addition, ILC lack the expression of specific lineage (Lin) markers (i.e. CD3 (T-cells), CD19 (B-cells), CD11b (neutrophils/eosinophils) and CD11c (dendritic cells/macrophages)), but express CD127 (IL-7Rα), the receptor for IL-7. We further refer to these CD45^+^ Lin^-^ CD127^+^ cell population as the pulmonary ILC population (Fig 1A).

To make a distinction between the different ILC subsets within the pulmonary ILC population, we used specific surface markers. Since NK cells could contaminate the ILC1 subset, we used the CD56 marker to exclude NK cells and characterized the non-toxic ILC1 subset as CD56^-^ IL12Rβ2^+^ pulmonary ILC (Fig 1B). The ILC2 subset was identified as pulmonary ILC that express the chemoattractant receptor-homologous molecule expressed on T_H_2 cells (CRTH2), in analogy with ILC2 that previously were detected in the upper airways (Fig 1C). Both ILC3 subsets, NCR^+^ ILC3 and NCR^-^ ILC3, were gated as CD117^+^ (c-kit) pulmonary ILC and a further distinction was made by NKp44, a natural cytotoxicity receptor (Fig 1D). Using these staining combinations, we were able to discriminate between the three ILC subsets in human lung tissue. Moreover, we demonstrated that our staining combination was specific, since the surface markers that were used to characterize a specific ILC subset, were not expressed on other ILC subsets (S3 Fig). Interestingly, inclusion of extra lineage markers (i.e. CD1a, CD14, CD34, CD123, TCRαβ, TCRγδ, BDCA2 and FcεR1) resulted in identical percentages of the different ILC subsets (S4 Fig).

### Intracellular staining of transcription factors in pulmonary ILC subsets

**Fig 2:**
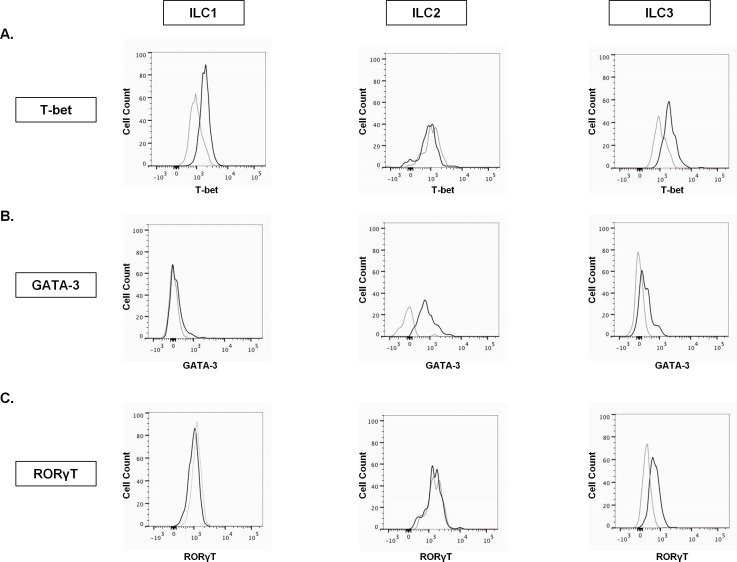
Intracellular staining of transcription factors in pulmonary ILC subsets. The developmental transcription factors were determined in the specific ILC subsets on single cell suspensions of digested human lung. **A,** Expression of T-bet (black line) versus isotype control (grey line) in the ILC1 (CD45^+^, Lin^-^ CD127^+^, CD56^-^, CRTH2^-^, CD117^-^), ILC2 (CD45^+^, Lin^-^, CD127^+^, CRTH2^+^), ILC3 (CD45^+^, Lin^-^, CD127^+^, CD117^+^) population. **B,** GATA-3 expression (black line) versus isotype control (grey line) in the different ILC subsets. **C,** Expression of RORγT (black line) versus isotype control (grey line) in ILC1, ILC2 and ILC3 population.

Besides characterization based on surface markers, also specific transcription factors can be used to characterize ILC subsets. For that purpose, we assessed the expression of key transcription factors within the different ILC subsets using intracellular flow cytometry. T-bet expression was detectable within the non-toxic ILC1 subset and – interestingly – also in the ILC3 subset, but not in the ILC2 subset (Fig 2A). The transcription factor GATA-3 was clearly expressed in the ILC2 subset (Fig 2B), whereas no to limited GATA-3 levels were found in the ILC1 and ILC3 subsets (Fig 2B). Finally, specific expression of RORγT was demonstrated in the ILC3 subset; which contrasts with the ILC1 and ILC2 subsets that were both negative for the transcription factor RORγT (Fig 2C).

### Intracellular cytokine production in the pulmonary ILC population

**Fig 3:**
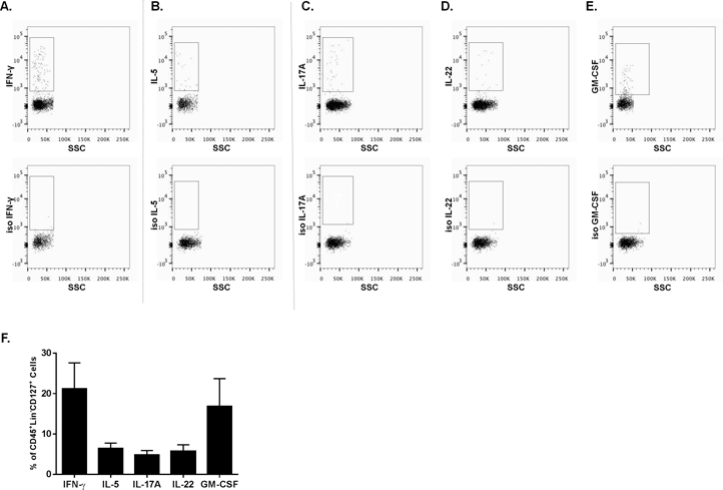
Intracellular cytokine production in the pulmonary ILC population. Several signature cytokines in pulmonary ILC (gated as CD45^+^, Lin^-^ CD127^+^ cells) were determined on single cell suspensions of digested human lung (n = 8). Since NK cells could contaminate the non-toxic ILC1 subset, CD56^+^ cells were excluded to investigate the IFN-γ production. For the production of these cytokines, lung cells were first stimulated for 15 hours with PMA/ionomycin (+ transport inhibitors). **A,** IFN-γ production in ILC. **B,** Production of IL-5 in the pulmonary ILC population. **C,** IL-17 production in ILC. **D,** ILC production of IL-22. **E,** Production of GM-CSF in the ILC population. The bottom panels represent the isotype controls of the specific cytokine staining. **F,** Frequency of IFN-γ, IL-5, IL-17A, IL-22 and GM-CSF positive cells within the ILC (CD45^+^, Lin^-^ CD127^+^) population (n = 8, mean ± SEM).

To assess signature cytokines via intracellular flow cytometry, we stimulated lung single cell suspensions for 15 hours with PMA/ionomycin (+ protein transport inhibitors). Due to the scarcity of individual ILC subsets in human lung, signature cytokines where examined in the total pulmonary ILC population (i.e. CD45^+^ Lin^-^ CD127^+^ cells). Additionally, since NK cells are able to produce type 1 cytokines, CD56 was used as an extra exclusion marker during the analyses of IFN-γ. We observed production of IFN-γ, indicative for ILC1 in the human lung tissue (Fig 3A). Similarly, we could identify type 2 cytokine production, specifically IL-5 expression, which suggested the presence of pulmonary ILC2 in the human lung tissue (Fig 3B). Finally, also cytokines produced by ILC3 such as IL-17A (Fig 3C), IL-22 (Fig 3D) and GM-CSF (Fig 3E) were seen within the pulmonary ILC population. Fig 3F shows the frequency of cytokine positive cells in the ILC population. Of note, unstimulated pulmonary ILC had no to limited basal cytokine expression (data not shown).

### ILC subsets in control subjects versus patients with COPD

**Fig 4:**
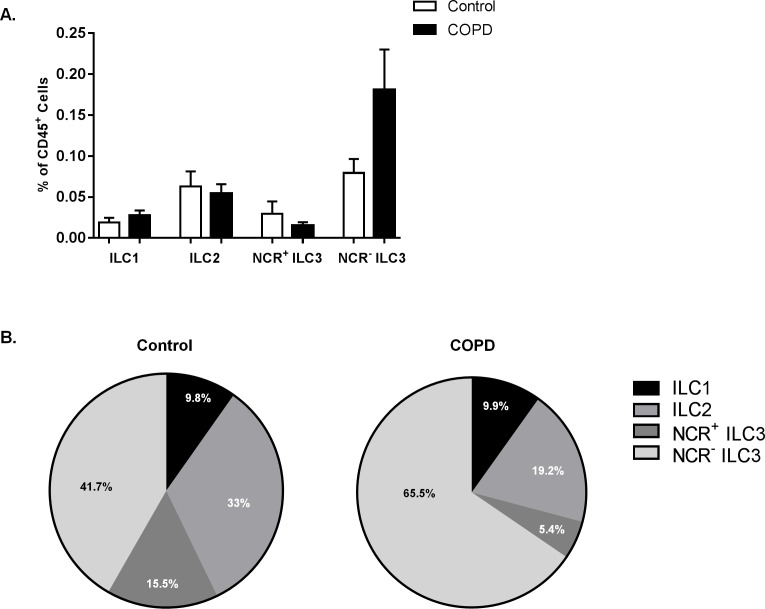
ILC subsets in control subjects versus patients with COPD. **A,** The frequency of ILC1 (CD45^+^, Lin^-^, CD127^+^, CD56^-^, IL12Rβ2^+^), ILC2 (CD45^+^, Lin^-^, CD127^+^, CRTH2^+^), NCR^+^ ILC3 (CD45^+^, Lin^-^, CD127^+^, CD117^+^, NKp44^+^) and NCR^-^ ILC3 (CD45^+^, Lin^-^, CD127^+^, CD117^+^, NKp44^-^) in digested human lung from control (n = 5) and COPD patients (n = 11) was determined by flow cytometry. ILC numbers were expressed as percentages (%) of the CD45^+^ population (mean ± SEM). **B,** Pie chart of the relative abundance of ILC1, ILC2, NCR^+^ ILC3 and NCR^-^ ILC3 subsets in control subjects and patients with COPD.

Using the above described surface markers, we quantified the percentages of ILC1, ILC2, NCR^+^ ILC3 and NCR^-^ ILC3 subsets in lung single cell suspensions. Since the relative abundance of pulmonary ILC subsets could be altered in diseases such as COPD, we investigated the frequency of the different ILC subsets in control subjects (n = 5) and in patients with COPD (n = 11). Although there were no significant differences observed in the relative abundance of the specific ILC subsets between the control and COPD group (probably due to low patient numbers) (Fig 4A), some interesting findings were found. In the control group, both ILC2 and NCR^-^ ILC3 were the most abundant ILC subsets. Interestingly, in patients with COPD the distribution of ILC tended to shift to a greater presence of NCR^-^ ILC3 compared with the other ILC subsets (Fig 4B). Moreover, when analysing cytokine positive ILC, we observed that IL-17A and IL-22 expressing ILC tended to increase in patients with COPD compared with controls, whereas IFN-γ and IL-5 expressing ILC were similar (S5 Fig)

## Discussion

**Fig 5:**
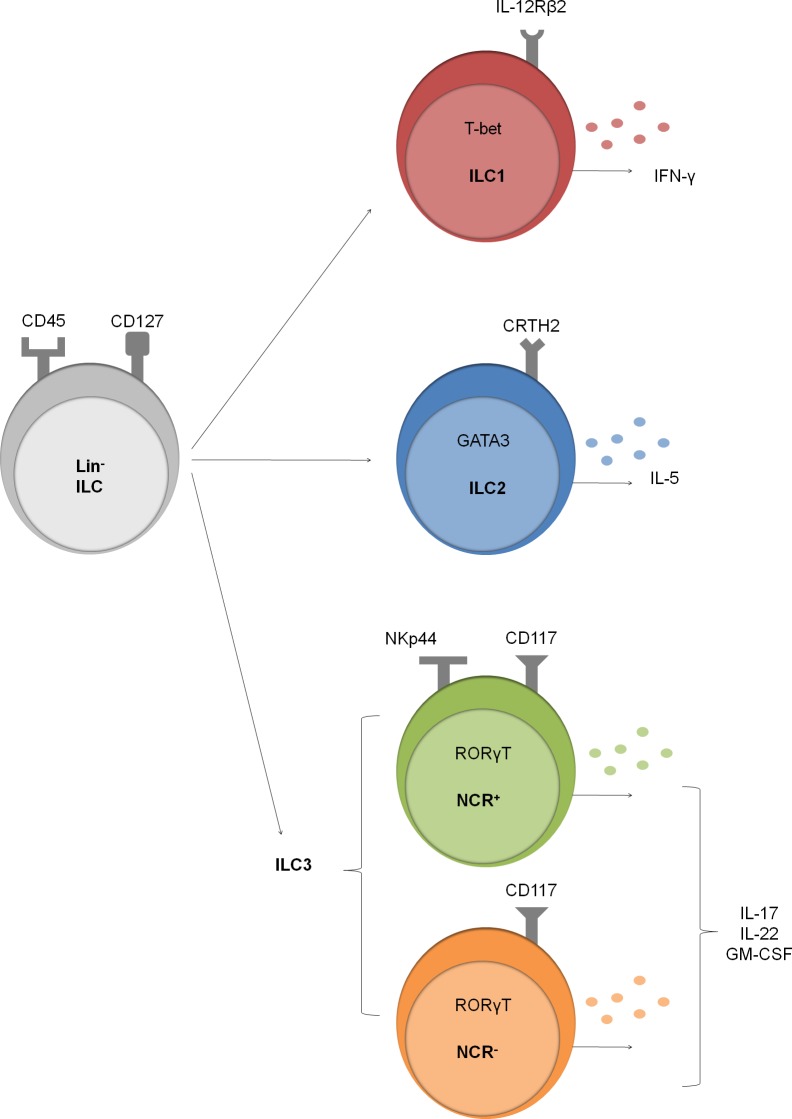
Overview of innate lymphoid cell subsets in human lung tissue. The presence of CD45^+^, Lin^-^ (i.e. CD3, CD19, CD11c, CD11b) and CD127^+^ ILC in pulmonary tissue was demonstrated. These ILC were further subdivided in a CD56^-^ IL12Rβ2^+^ ILC1 subset, CRTH2^+^ ILC2 subset, CD117^+^ NKp44^+^ (NCR^+^) ILC3 subset and CD117^+^ NKp44^-^ (NCR^-^) ILC3 subset. Further, expression of signature transcription factors (i.e. T-bet, GATA-3 and RORγT) within the specific ILC subset and cytokine production (i.e. IFN-γ, IL-5, IL-17A, IL-22 and GM-CSF) within the pulmonary ILC population was demonstrated.

We demonstrate the presence of all ILC subsets in human lung (Fig 5). Using specific surface markers and key transcription factors, we characterized the ILC1, ILC2, NCR^+^ ILC3 and NCR^-^ ILC3 subsets in human lung single cell suspensions by multi-color flow cytometry. In addition, we assessed the production of signature cytokines in the pulmonary ILC population. Furthermore, our data suggest that the frequency of NCR^-^ ILC3 tended to increase in patients with COPD.

To characterize ILC subsets in human tissues, specific surface markers have been proposed in the literature. In gut mucosal tissue, a higher fraction of the IL12Rβ2 transcript could be detected in the ILC1 population [15]. Using this specific marker, as well as CD56 to exclude contaminating NK cells [3,16], we identified lung ILC1 as CD45^+^ Lin^-^ CD127^+^ CD56^-^ IL12Rβ2^+^ cells. However, it should be noted that some controversy still exists in the characterization of the (human and mouse) ILC1 subset. At least in the gut, an additional intraepithelial CD127^low^ CD103^+^ ILC1 subset has been discovered, that would be the equivalent of cytotoxic CD8^+^ T-cells [4]. Recently, such a CD127^low^ CD103^+^ ILC1 subset was also found in human lung tissue [11]. Compatible with research findings in the gut and nasal polyps [10], we identified pulmonary ILC2 as CRTH2^+^ cells within the pulmonary ILC population. ILC3 subsets in the gut and skin were distinguished based on NCR (NKp44) [17,18]. Accordingly, in human lung specimens, we show the presence of both NCR^+^ ILC3 and NCR^-^ ILC3 within the CD117^+^ pulmonary ILC. One should however be aware that the NCR^-^ ILC3 subset remains a heterogeneous population that also contains LTi-cells.

In addition to the characterization based on surface markers, the expression of developmental transcription factors is an important feature to identify ILC subsets. We demonstrated a clear expression of GATA-3 in the ILC2 subset and RORγT in the ILC3 subset, which suggests that our staining based on surface markers adequately discriminates between the ILC subsets. Of interest, besides T-bet expression in the ILC1 subset, we also demonstrated a high T-bet expression in the ILC3 subset. It was previously shown that, upon stimulation with IL-12, intestinal NCR^-^ ILC3 could lose their RORγT expression and upregulate T-bet, suggesting that ILC3 can differentiate into ILC1 [15,19]. Our observed T-bet signal in the ILC3 subset could therefore indicate a plasticity of human pulmonary ILC3, although this requires further investigation.

Upon stimulation, ILC are able to produce several effector cytokines. We observed expression of IFN-γ, IL-5, IL-17A and IL-22 in the pulmonary ILC population, indicative for the presence of activated ILC1, ILC2, NCR^+^ ILC3 and NCR^-^ ILC3. In addition, we also observed GM-CSF production which would be interesting to explore further, since RORγT^+^ ILC3 were able to produce GM-CSF in the mouse intestine which contributed to T-cell homeostasis [20,21]. However, it should be emphasized that the cytokine expression was investigated in the total pulmonary ILC population. Assessment of cytokine production by the specific pulmonary ILC subsets could provide additional functional insights. Furthermore, it would be worthwhile to investigate the production of amphiregulin by human pulmonary ILC2 in future experiments. At least in mice, it has been shown that besides type 2 cytokines, ILC2 can produce amphiregulin, an epidermal growth factor that has been implicated in wound healing and tissue (lung) remodeling [5].

Depending on the tissue, the composition of human ILC subsets can differ [4]. In healthy skin, for instance, higher numbers of ILC2 and NCR^-^ ILC3 were found [17], whereas NCR^+^ ILC3 are the most abundant ILC subset in the gut [22]. The number of ILC subsets in our control subjects is relatively low, but is in line with the number of ILC2 that was previously observed in non-inflamed nose tissue [10]. Further, it has been shown that the relative abundance of the ILC subsets can depend on the disease state, such as in Crohn’s disease, psoriasis and chronic rhinosinusitis [10,15,17]. Although there were no statistical differences between the control and COPD group, a trend to a higher relative abundance of NCR^-^ ILC3 could be observed in patients with COPD. It should however be noted that a small number of patients was investigated, and that our findings need to be confirmed in a larger study population.

In COPD patients, a higher number of lymphoid follicles was found compared to controls that correlated with disease severity [23,24]. Importantly, IL-17A and IL-22, which are produced by NCR^-^ ILC3, are crucial in the formation of lymphoid follicles [25,26]. It would therefore be interesting to investigate the role of the increased NCR^-^ ILC3 subset in lymphoid follicle formation in patients with COPD. Furthermore, as lymphoid follicles are also observed in other chronic pulmonary diseases (i.e. pulmonary fibrosis, bronchiectasis, follicular bronchiolitis, lymphoid interstitial pneumonia) [24], it would be worthwhile to also examine the relative abundance of NCR^-^ ILC3 in these disease settings. Besides being involved in the formation of lymphoid follicles, NCR^-^ ILC3 could also contribute to the persistent inflammation in COPD patients by pro-inflammatory cytokine production. Alternatively, it could be that the accumulation of NCR^-^ ILC3 in COPD is associated with host protective immunity, in response to bacterial respiratory tract infections which occur frequently in patients with COPD [24]. Further functional studies that address the role of NCR^-^ ILC3 in COPD are therefore warranted. Very little is known about the (protective and pathological) role of ILC3 in lung homeostasis as well as in the context of disease. Recently, NCR^-^ ILC3 were found to accumulate in human non-small cell lung cancer tissue, where they might contribute to the formation of protective tumour-associated tertiary lymphoid structures [11].

Untill now, studies in the respiratory system have mainly focused on the ILC2 subset. Although several animal studies have described an important role for ILC2 in helminth infections [27], allergic airway inflammation [28–31] and airway hyperresponsiveness [32,33], the function of ILC2 in the human lung remains incompletely studied. To date, increased numbers of ILC2 in nasal polyps from patients with chronic rhinosinusitis have been demonstrated [9,10], suggesting a role for ILC2 in eosinophilic inflammation in the upper airways. In addition, genes discovered in genome-wide association studies of asthma (RORα, IL-13, IL-33, IL-1RL1; which are all related to ILC2) suggest a key role for ILC2 in asthma [34]. We recently hypothesized that ILC2 could have an important role in non-allergic eosinophilic airway inflammation [35]. In support of this, research in an experimental model of asthma has shown that ILC2 are highly corticosteroid resistant [36], which could explain why severe eosinophilic asthmatics are relatively corticosteroid resistant [35].

In summary, based on expression of surface markers and key transcription factors, we demonstrated that ILC1, ILC2, NCR^+^ ILC3 and NCR^-^ ILC3 subsets are present in the human lung. In addition, pulmonary ILC were able to produce signature cytokines upon stimulation. Of interest, we showed that pulmonary NCR^-^ ILC3 tended to accumulate in the lung of COPD patients, although this should be confirmed in a larger study population. The functional role of ILC subsets in lung homeostasis and pulmonary diseases however remains to be fully elucidated. Further research on the function of ILC subsets is therefore needed to address whether ILC are possible targets for new therapeutics in (chronic) pulmonary diseases.

## Supporting Information

S1 Fig Process of making single cell suspensions of lung resection specimen. Lung tissue was obtained from patients who underwent a surgical lung resection. Tissue was cut into fine pieces and digested for 45 minutes at 37°C in digestion medium supplemented with collagenase type 2 and DNase I. Next, EDTA was added to stop the digestion and the lung cells were filtered through a 40-μm cell strainer. Pulmonary mononuclear cells were isolated with Ficoll-Paque^TM^ plus. Finally, cells were subjected to red blood cell (RBC) lysis and stained for flow cytometry.
(TIF)

S2 Fig Fluorescence minus one (FMO) controls to set up an adequate ILC gating strategy. FMO controls were used on single cell suspensions of digested human lung and analyzed by flow cytometry. FMO controls contain every stain in the ILC panel except for that specific fluorochrome that was investigated. This figure shows how the gate for the specific ILC subsets (Fig 1) was set as compared to the different FMO controls.
(TIF)

S3 Fig Specificity of ILC staining combination. **A,** Expression of CRTH2, CD117 in the pulmonary ILC1 population. **B,** Analyses of the surface markers IL12Rβ2, CD117 in the ILC2 subset. **C,** Expression of IL12Rβ2, CRTH2 in the pulmonary ILC3 subset.
(TIF)

S4 Fig Inclusion of extra lineage markers in the ILC cocktail. Inclusion of additional lineage markers in the ILC cocktail was assessed by flow cytometry on single cell suspensions of digested human lung. Frequencies of ILC1, ILC2 and ILC3 in digested human lungs using our ‘classical’ lineage mix (i.e. CD3, CD19, CD11c, CD11b) (filled symbols) and the lineage mix with extra markers (i.e. CD3, CD19, CD11c, CD11b, CD1a, CD14, CD34, CD123, TCRαβ, TCRγδ, BDCA2 and FcεR1) (open symbols) are shown. n = 3.
(TIF)

S5 Fig Frequency of cytokine positive ILC in control subjects and patients with COPD. Frequency of IFN-γ, IL-5, IL-17A, IL-22 positive ILC (gated as CD45^+^, Lin^-^, CD127^+^ cells) in digested human lung of control subjects (n = 2) and COPD patients (n = 6) was determined by intracellular flow cytometry staining (mean ± SEM).
(TIF)
