# Supplementary figures and images for: Characterization and Quantification of Innate Lymphoid Cell Subsets in Human Lung
Source: PLoS One. 2016 Jan 4;11(1):e0145961. doi: 10.1371/journal.pone.0145961 (PMC4699688; doi:10.1371/journal.pone.0145961)

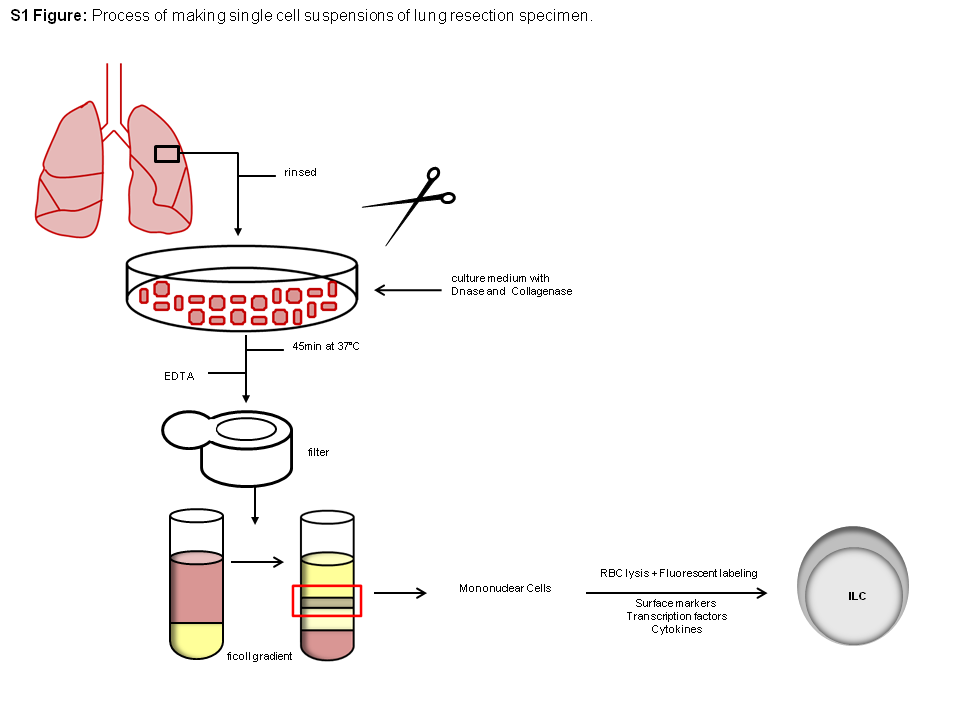

Supplement: S1 Fig — Lung tissue was obtained from patients who underwent a surgical lung resection. Tissue was cut into fine pieces and digested for 45 minutes at 37°C in digestion medium supplemented with collagenase type 2 and DNase I. Next, EDTA was added to stop the digestion and the lung cells were filtered through a 40-μm cell strainer. Pulmonary mononuclear cells were isolated with Ficoll-PaqueTM plus. Finally, cells were subjected to red blood cell (RBC) lysis and stained for flow cytometry. (TIF) [file pone.0145961.s001.tif]

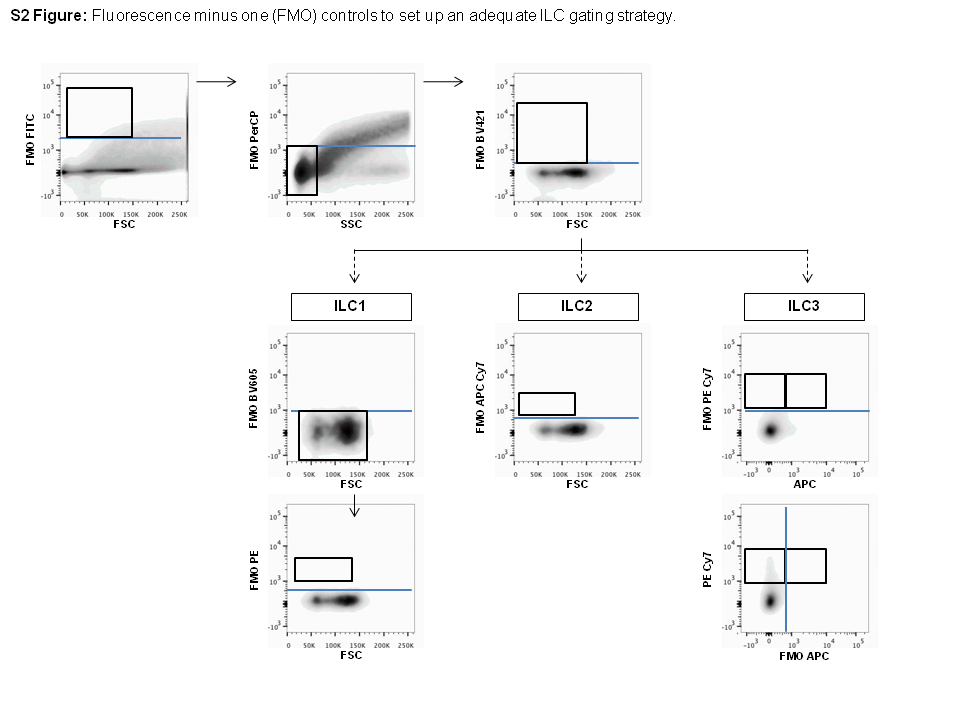

Supplement: S2 Fig — FMO controls were used on single cell suspensions of digested human lung and analyzed by flow cytometry. FMO controls contain every stain in the ILC panel except for that specific fluorochrome that was investigated. This figure shows how the gate for the specific ILC subsets (Fig 1) was set as compared to the different FMO controls. (TIF) [file pone.0145961.s002.tif]

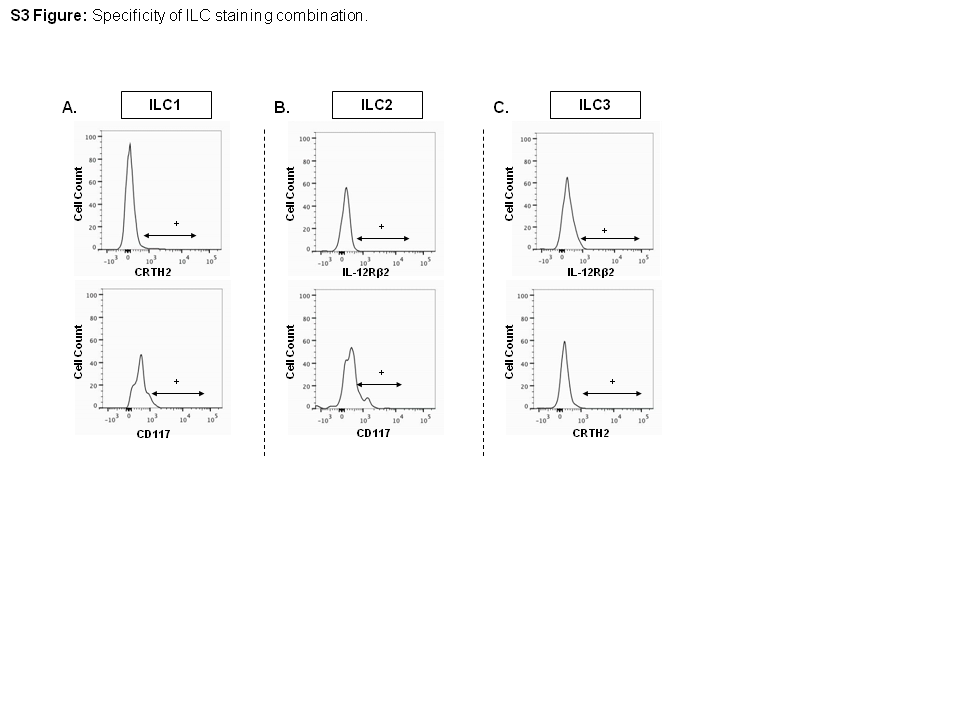

Supplement: S3 Fig — A, Expression of CRTH2, CD117 in the pulmonary ILC1 population. B, Analyses of the surface markers IL12Rβ2, CD117 in the ILC2 subset. C, Expression of IL12Rβ2, CRTH2 in the pulmonary ILC3 subset. (TIF) [file pone.0145961.s003.tif]

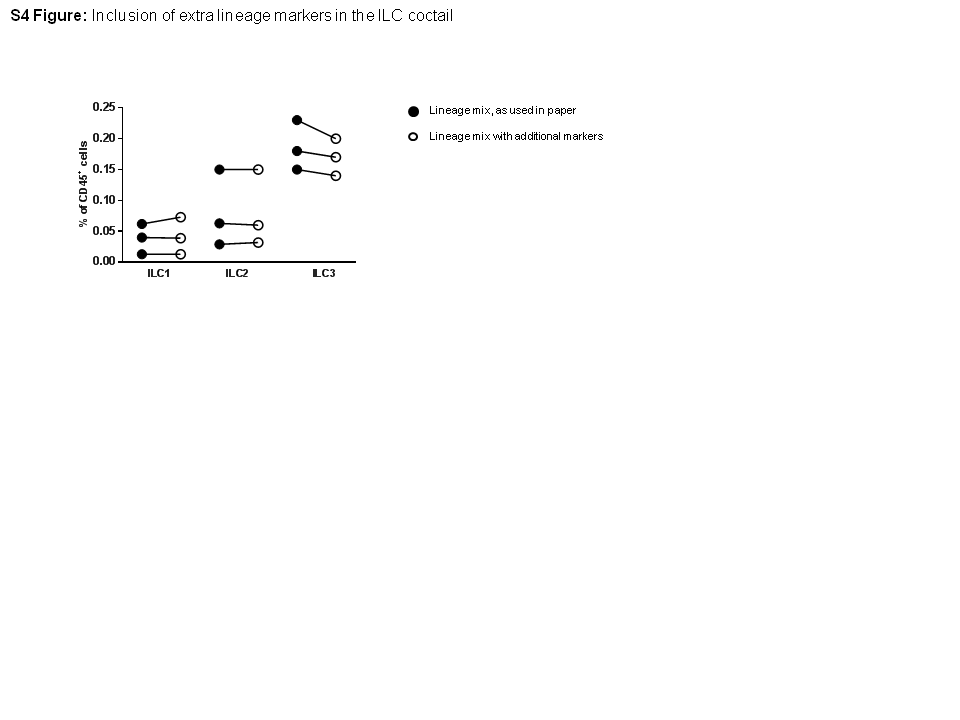

Supplement: S4 Fig — Inclusion of additional lineage markers in the ILC cocktail was assessed by flow cytometry on single cell suspensions of digested human lung. Frequencies of ILC1, ILC2 and ILC3 in digested human lungs using our ‘classical’ lineage mix (i.e. CD3, CD19, CD11c, CD11b) (filled symbols) and the lineage mix with extra markers (i.e. CD3, CD19, CD11c, CD11b, CD1a, CD14, CD34, CD123, TCRαβ, TCRγδ, BDCA2 and FcεR1) (open symbols) are shown. n = 3. (TIF) [file pone.0145961.s004.tif]

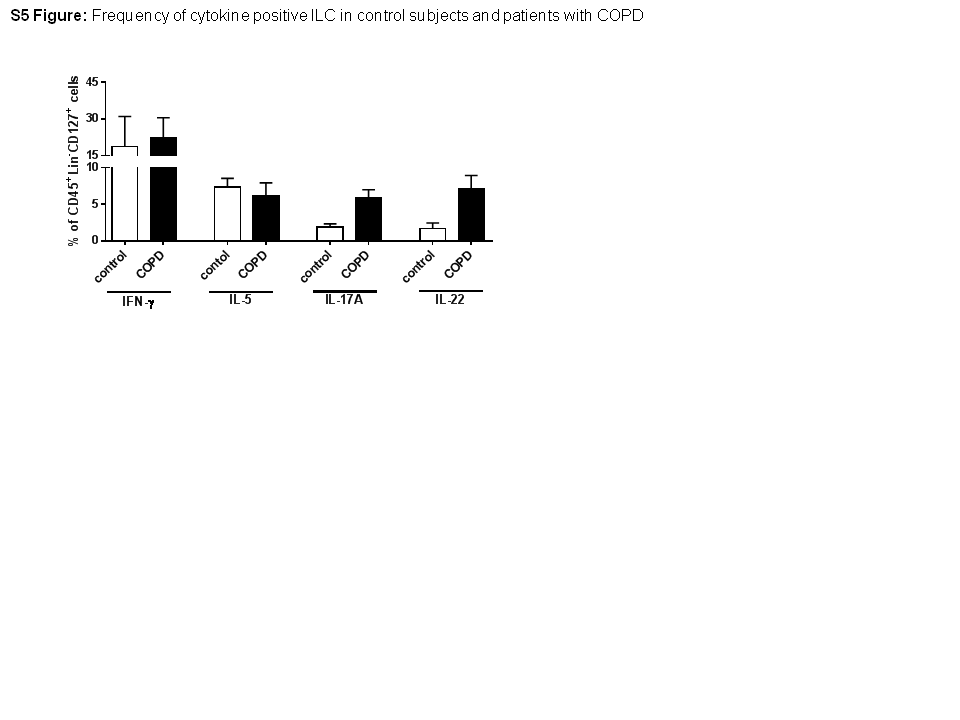

Supplement: S5 Fig — Frequency of IFN-γ, IL-5, IL-17A, IL-22 positive ILC (gated as CD45+, Lin-, CD127+ cells) in digested human lung of control subjects (n = 2) and COPD patients (n = 6) was determined by intracellular flow cytometry staining (mean ± SEM). (TIF) [file pone.0145961.s005.tif]
